# Adhesion and Invasion of Gastric Mucosa Epithelial Cells by *Helicobacter pylori*

**DOI:** 10.3389/fcimb.2016.00159

**Published:** 2016-11-22

**Authors:** Ying Huang, Qi-long Wang, Dan-dan Cheng, Wen-ting Xu, Nong-hua Lu

**Affiliations:** ^1^Department of Gastroenterology, The First Affiliated Hospital of Nanchang UniversityNanchang, China; ^2^Department of General Surgery, Tianjin Haihe HospitalTianjin, China

**Keywords:** *Helicobacter pylori*, adhesion, invasion, chronic infection, mechanism

## Abstract

*Helicobacter pylori* is the main pathogenic bacterium involved in chronic gastritis and peptic ulcer and a class 1 carcinogen in gastric cancer. Current research focuses on the pathogenicity of *H. pylori* and the mechanism by which it colonizes the gastric mucosa. An increasing number of *in vivo* and *in vitro* studies demonstrate that *H. pylori* can invade and proliferate in epithelial cells, suggesting that this process might play an important role in disease induction, immune escape and chronic infection. Therefore, to explore the process and mechanism of adhesion and invasion of gastric mucosa epithelial cells by *H. pylori* is particularly important. This review examines the relevant studies and describes evidence regarding the adhesion to and invasion of gastric mucosa epithelial cells by *H. pylori*.

## Introduction

*Helicobacter pylori* is a gram-negative, flagellated, microaerophilic bacterium that selectively colonizes the gastric mucosa. *H. pylori* is one of the most common infectious agents worldwide, and approximately 50% of the world's population is estimated to be infected (Marshall and Warren, 1983, 1984). Although the detailed transmission route of *H. pylori* remains uncertain, an oral-oral or fecal-oral route during childhood is thought to be the most plausible method of human-to-human transmission (Goh et al., 2011). Once established, *H. pylori* has no significant bacterial competitors (Peek and Blaser, 2002). The prevalence of *H. pylori* infection varies widely by geographic area, age, race, and socioeconomic status (SES), and developing appear to have higher infection rates than developed countries (Brown, 2000). Indeed, the prevalence exhibits country-to-country variation, with values as low as 15.4% in Australia to values as high as 90% in developing countries such as Iran (Moujaber et al., 2008; Hosseini et al., 2012; Siao and Somsouk, 2014). *H. pylori* is unique in that the bacterium can persist for decades in the harsh stomach environment, where it damages the gastric mucosa and alters the pattern of gastric hormone release, thereby affecting gastric physiology (Wang et al., 2014). The slow development of cancer known as Correa's cascade (Correa, 1992) includes a series of intermediate stages (precancerous lesions) before malignancy *per se* occurs. These precancerous lesions occur in the following order: gastritis, atrophy, intestinal metaplasia (IM), and eventually dysplasia. *H. pylori* represents the most significant risk factor for gastric malignant tumors (Wang et al., 2014). Gastric cancer (GC) is an insidious disease, with symptoms that often manifest at an advanced stage, a time when the few remaining therapeutic options have low efficiency (Boreiri et al., 2013). Approximately 10% of infected individuals develop severe gastric lesions, such as those in peptic ulcer disease; 1–3% progress to GC, with a low 5-year survival rate (Cirak et al., 2007), and 0–1% develop mucosa-associated lymphoid tissue (MALT) lymphoma (Noto and Peek, 2012; Parreira et al., 2013; Wang et al., 2014). Compared with uninfected individuals, individuals infected with *H. pylori* are estimated to have a 2–8-fold increased risk of developing GC (Huang et al., 1998; Eslick et al., 1999; Helicobacter and Cancer Collaborative Group, 2001; Kamangar et al., 2006; Wang et al., 2007), and the International Agency for Research on Cancer (IARC) has classified *H. pylori* as a class I carcinogen (IARC Working Group on the Evaluation of Carcinogenic Risks to Humans, 1994). However, the inability of the immune system to clear *H. pylori* infection is not well described. Furthermore, the mechanisms controlling the induction and maintenance of *H. pylori*-induced chronic inflammation are only partly understood.

In the past, *H. pylori* was considered to be a non-invasive bacterium that generally only adhered to gastric mucosa epithelial cells or survived in the gastric lumen. In contrast, recent studies have shown that *H. pylori* is invasive, and it is now regarded as a special intracellular pathogen (Petersen and Krogfelt, 2003; Dubois and Borén, 2007). *H. pylori* microcolonies form on the surface of the cell membrane, and this area then becomes the microenvironment for bacterial reproduction (Tan et al., 2009). *H. pylori* can invade cells and replicate to reproduce and complete an entire biological cycle by cell division (Chu et al., 2010). Nonetheless, the mechanism of invasion remains unclear, and studies to date have mainly concentrated on receptor-mediated endocytosis and a tyrosine kinase-dependent process (Evans et al., 1992; Birkness et al., 1996; Su et al., 1999; Kwok et al., 2002). A phagosome forms after *H. pylori* invades gastric epithelial cells; the bacterium then exits cells to colonize again while conditions are suitable and repeatedly infects cells. These findings suggest that invasion might play an important role in disease induction, immune escape, and chronic infection (Kwok et al., 2002; Dubois and Borén, 2007; Jang et al., 2013). In this “cellular internalization” process, a bacterium specifically binds to a host cell receptor and enters the cytoplasm via phagocytic vacuoles formed through cell membrane invagination. Therefore, to provide insight into the pathogenic mechanism of *H. pylori*, this article will review relevant studies and describe evidence for the adhesion and invasion of gastric mucosa epithelial cells by this bacterium.

## Host cell adhesion by *H. pylori*

Within the gastric mucus layer, bacteria can be found relatively close to the gastric lumen or deep within the gastric glands, and these microbes can either swim freely (Hazell et al., 1986; Schreiber et al., 2004; Celli et al., 2009) or attach to gastric epithelial cells (Hessey et al., 1990). To achieve successful engraftment, the most important step in *H. pylori* infection, the bacterial cells must survive under various unfavorable conditions, such as exposure to pepsin and an extremely low pH. Engraftment occurs primarily when adhesion molecules or other molecules on the surface of *H. pylori* bind to mucins, allowing the bacterial cells to colonize the gastric mucosa epithelium. This event triggers expression of several bacterial genes, including some that encode virulence factors and protect the pathogen from clearance mechanisms, such as liquid flow, peristaltic movement or shedding of the mucous layer (Kim et al., 2004). This process includes at least two steps: (1) *H. pylori* quickly moves through the mucus layer to the surface of the gastric mucosa, which has a relatively neutral pH, under the aegis of the pH buffer mechanism; and (2) *H. pylori* firmly adheres to gastric mucosa epithelial cells via the outer membrane protein (OMP).

## Mucins

Mucins, which are high molecular weight, heavily glycosylated glycoproteins secreted by epithelial cells, play a key role in the adhesion process. Mucins are located on the surface of the stomach cavity and are the main components of the gastric mucosa epithelial mucus layer. At this location, mucins form a barrier in the mucosal defense system that protects gastric epithelial cells against chemical, enzymatic, microbial, and mechanical damage. Mucin-1 (MUC-1), Mucin-5AC (MUC-5AC), and Mucin-6 (MUC-6) are expressed in the gastric mucosa of normal adults, and the genes encoding these mucins are located on different chromosomes. The main structures formed by mucins are protein scaffolds based on the core peptide, the typical structure of which is a variable number of tandem repeats (VNTRs) rich in serine, threonine and proline, which are potential glycosylation sites. One study has to date demonstrated that MUC-5AC and MUC-6 are separated in the gastric mucus gel, resulting in a layered linear array (Ho et al., 2004). The former is mainly found on the surface and bottom of the mucus gel, and the latter is present among the various layers. This natural stratification of mucins increases the viscosity of gastric mucosa epithelial mucus gel and provides an independent system to completely protect gastric mucosa epithelial cells. Therefore, *H. pylori* must pass through mucins to successfully adhere to to host cells.

## Ureases and motility

Both urease and flagella-mediated, chemosensory-directed motility are essential and relevant to the process of colonization (Eaton et al., 1991, 1996; Nakamura et al., 1998; Rolig et al., 2012). *H. pylori* secretes a large amount of urease, and the surface-bound enzyme catalyzes hydrolysis of urea to generate ammonia and bicarbonate, which are then released into the cytosol and periplasm, forming a neutral environment around the bacterial cells (Weeks et al., 2000). This process decreases the viscosity and elastic modulus of the mucus layer, which changes from a gel to a viscous solution with increasing pH, thereby facilitating the passage of the bacteria through the mucus. Ammonia protects the metabolic activity of *H. pylori*, which remains at 50–60% of its normal activity level in the highly acidic environment of pH 2.5 (Celli et al., 2009; Follmer, 2010). Microscopy studies of the motility of *H. pylori* in the gastric mucosa at acidic and neutral pH values in the absence of urea showed that the bacteria swim freely at high pH but are highly constrained at low pH (Celli et al., 2009). From a hydrodynamics viewpoint, the shape of the *H. pylori* cell can also affect its swimming speed. Previous studies have suggested that *H. pylori* possesses numerous long flagella that may allow the cell to swim through the sticky viscoelastic mucus gel in a manner similar to how a screw passes through a cork (Berg and Turner, 1979; Karim et al., 1998). Swimming speeds also decrease with increasing viscosity of the polymer solution (Worku et al., 1999). The results of Martínez et al. (2016) are consistent with the above observations. On this basis, these authors provide an in-depth, quantitative analysis of *H. pylori*'s natural variation in helical cell and flagellum morphology, indicating that both cell shape and flagellum number independently affect swimming speed in viscous environments, with flagellum number contributing to a greater degree. In addition, as for other commensal microbes that colonize a host over long periods of time, *H. pylori* must sense and integrate many signals emanating from the gastric epithelium that attract the microbes to the cell surface for colonization and persistence. This ability to move in response to chemical cues, i.e., chemotaxis, is determined by the core signaling complex proteins CheW, CheA, and CheY (Beier et al., 1997; Foynes et al., 2000; Pittman et al., 2001). *H. pylori* also has three membrane-bound chemoreceptors, TlpA, TlpB, and TlpC, and one cytoplasmic chemoreceptor, TlpD (Lertsethtakarn et al., 2011). Rolig et al. (2012) found that different regions of the stomach contain unique chemotactic signals. For example, in the corpus, *H. pylori* utilizes chemotaxis for initial localization but not for subsequent growth. In contrast, in the antrum and the corpus-antrum transition zone, chemotaxis does not help initial colonization but does promote subsequent proliferation. The main chemoreceptor that allows *H. pylori* to thrive in the antrum is TlpD, with the other chemoreceptors playing minor roles. Thus, chemotaxis may be necessary to locate the antrum or to maintain colonization at this site. Indeed, chemotaxis of *H. pylori* toward the urea present on the epithelial cell surface may also be crucial for survival in the stomach (Nakamura et al., 1998). It was recently demonstrated that *H. pylori* swims toward injured epithelia, suggesting that the bacterial cells are attracted to host-derived molecules (Aihara et al., 2014). The main bacterial chemoreceptor responsible for this chemoattraction is TlpB, and urea has been identified as the host metabolite that attracts *H. pylori* (Huang et al., 2015). In addition, Huang et al. (2015) revealed a function for *H. pylori* urease in facilitating sensitive detection of urea at concentrations as low as 50 nanomolar. Therefore, *H. pylori* has evolved a sensitive urea chemodetection and destruction system involving a high-affinity chemoreceptor-ligand interaction that functions at the nanomolar concentrations created locally by urease, allowing the bacterium to dynamically and locally modify the host environment to locate the epithelium. Overall, we believe that the ability of *H. pylori* to bore through the mucus gel might be achieved in two ways: flagella and chemotaxis provide some contribution, but changes in the rheological properties of the environment are also important.

## Adhesins

Adhesins, which attach to the surface of the bacterium, play a vital role because they can recognize the structures of glycans expressed in the gastric mucosa, and *H. pylori* must swim through the mucus gel (Ilver et al., 1998; Mahdavi et al., 2002). Using adhesins, *H. pylori* can identify peptidoglycans on the surface of gastric epithelial cells, molecules that are mainly found on mucins in the gastric mucus gel. Furthermore, any *H. pylori* cell that does not adhere to an epithelial cell would be quickly removed from the epithelial cell surface and the mucus gel. At present, it is generally believed that OMPs are the main adhesins of *H. pylori*. Among these adhesins, blood group antigen-binding adhesion (BabA) can identify the difucosylated ABO/Lewis b (LeB) antigen present on red blood cells and gastrointestinal mucosa epithelial cells. In normal gastric tissue, LeB-binding strains always bind to MUC-5AC- and LeB-positive epithelial cells (Van de Bovenkamp et al., 2003). Moreover, the BabA-Leb interaction is important not only for *H. pylori* to adhere to the stomach surface but also to anchor the bacterial secretion system to the host cell surface for effective injection of bacterial factors into the host cell cytosol, which is the cause of clinical outcomes. In other words, once BabA binds to LeB, Type IV secretion system (TFSS)-dependent host cell signaling is triggered to induce the transcription of genes that enhance inflammation, intestinal metaplasia development, and associated precancerous transformation (Ishijima et al., 2011). Another adhesin is sialic acid-binding adhesion (SabA), which can bind to the antigens Lewis X (sLex) and Lewis a (sLea). Lewis antigens are common in infected and inflamed gastric mucosa (Lindén et al., 2008). SabA expression can rapidly respond to changing conditions in the stomach or in different regions of the stomach that permit *H. pylori* to adapt to varying microenvironments or host immune responses to ensure long-term colonization and infection (Goodwin et al., 2008). Accordingly, SabA-mediated adherence is positively correlated with the sLex concentration *in vitro* (Lindén et al., 2004). During persistent infection and chronic inflammation, *H. pylori* triggers an alteration in the glycosylation patterns in the gastric mucosa, including upregulation of inflammation-associated sLex antigens, and *H. pylori* is likely to adhere to the gastric mucosa with SabA. In addition, SabA interacting with the sLex antigen can enhance *H. pylori* colonization in patients with weak or no Leb expression (Yamaoka, 2008). SabA production is indeed reported to be associated with severe intestinal metaplasia, gastric atrophy, and the development of gastric cancer (Yamaoka et al., 2006). However, de Klerk et al. (2016) also found certain *Lactobacillus* strains could inhibit SabA expression at the transcriptional level by releasing an effector molecule into the medium, further affecting *H. pylori* binding capacity and thereby reducing its adherence. In particular, *Lactobacillus*, a well-known member of the normal microbiota in the human gastrointestinal tract, has always been studied in relation to *H. pylori* but largely as a possible supplement for antibiotic treatment (Patel et al., 2014), and interference with *H. pylori* colonization may contribute to the molecular mechanism for this association (de Klerk et al., 2016). AlpA and AlpB as OMPs also play a role in adherence to host cells and tissues (Odenbreit et al., 1999, 2002a). The alpAB locus, which is ubiquitously present in *H. pylori* strains, is expressed during infections (Rokbi et al., 2001; Odenbreit et al., 2009), and alpAB mutants are defective in adherence to human gastric tissue sections (Odenbreit et al., 1999). One study demonstrated that laminin is the host receptor of both AlpA and AlpB, and *H. pylori* deficient in these factors causes more severe inflammation than the isogenic wild-type strain in gerbils (Senkovich et al., 2011). The reason may be that the alpAB locus influences host cell signaling and cytokine production (Odenbreit et al., 2002a,b; Loke et al., 2007; Lu et al., 2007). *H. pylori* CagL protein is a specialized adhesin that is targeted to the pilus surface, where it binds to and activates integrin alpha5beta1 receptor on gastric epithelial cells through an arginine-glycine-aspartate motif. This interaction can mediate receptor-dependent delivery of CagA into gastric epithelial cells and may assist in certain intracellular signaling events.(Kwok et al., 2007; Barden et al., 2013). Current evidence suggests that MUC-1 in fact inhibits *H. pylori* colonization *in vitro* and *in vivo* (McGuckin et al., 2007; Lindén et al., 2009). Lindén et al. (2009) showed that MUC1 could inhibit *H. pylori* binding to epithelial cells, a process that occurs through both BabA and SabA. When the pathogen fails to bind to MUC1, the mucin sterically inhibits adhesion to other potential cell surface ligands. However, when the pathogen does bind to MUC1, the extracellular domain of the mucin is released from the epithelial surface and acts as a releasable decoy to prevent prolonged adherence. *H. pylori* also can modulate host cell glycosylation patterns to enhance adhesion. A highly pathogenic strain was able to alter expression of β3GlcNAcT5 (β3GnT5), a GlcNAc transferase essential for the biosynthesis of Lewis antigens, to increase sLex expression and *H. pylori* adhesion in human gastric carcinoma cell lines (Marcos et al., 2008). Furthermore, although the urease and alkyl hydroperoxide reductase (AhpC) located on the surface of *H. pylori* are not OMPs, these enzymes still have good affinity for stomach mucins because they are components of the membrane (Nilsson et al., 2000). Loke et al. (2007) confirmed that both polysaccharides and mucin could bind to AhpC and UreA, so polysaccharides may function as a potential anti-adhesive agent against *H. pylori* colonization of gastric mucin by competing for mucin-binding sites. This result indirectly indicated the probable role of these two *H. pylori* proteins in colonization. Other adhesins, such as HopZ and OipA, are also involved in the adhesion process, though the specific ligands need to be further clarified.

## Adherens junctions

In the gastric mucosa, the basement membrane is separated from the gastric lumen by only a single cell layer, and *H. pylori* can swim freely in the mucus layer in close contact with the epithelial cells, preferentially at the apical side of intercellular contacts (Hazell et al., 1986; Necchi et al., 2007; Tan et al., 2009). Any damage to the epithelial cell layer will expose extracellular matrix proteins, and disruptions of tight junctions by *H. pylori* will also allow the bacterial cells access to the basement membrane (Necchi et al., 2007). *H. pylori* targeting of adherens junctions may be beneficial for colonization and persistence at the host epithelial surface, and it may also cause abnormal receptor activation and stimulation of signaling pathways involved in inflammation, proliferation, migration, and invasion, which will have detrimental consequences and result in disease development (Costa et al., 2013).

## Cellular invasion of *H. pylori*

### Observation *in Vivo* and *in Vitro*

Adhesion is an important step in *H. pylori* internalization, and invasion has been confirmed in a variety of samples (Table 1). Some *in vivo* studies demonstrate that *H. pylori* can invade gastric mucosa lesions in different stages. For instance, invasion was observed in patients with gastritis, ulcers, precancerous lesions and gastric cancer, and the bacteria were able to invade epithelial cells of the stomach and duodenum, parietal cells and immune cells, and even the lamina propria (Noach et al., 1994; el-Shoura, 1995; Papadogiannakis et al., 2000; Semino-Mora et al., 2003; Dubois and Borén, 2007; Necchi et al., 2007; Ozbek et al., 2010). Animals, such as mice, are also susceptible to *H. pylori* invasion (Oh et al., 2005), and *in vitro* studies have shown that *H. pylori* can enter cells. Although *H. pylori* is a common pathogenic bacterium of the human digestive system, invasion is not limited to cell lines that are closely related to gastric epithelial cells, such as AGS, MKN45, and SGC-7901. Indeed, invasion has been confirmed in the Huh7, HEp-2, and HeLa cell lines, among others (Björkholm et al., 2000; Amieva et al., 2002; Kwok et al., 2002; Terebiznik et al., 2006; Zhang et al., 2007; Ito K. et al., 2008).

**Table 1:** **Overview of related studies on ***H. pylori*** invasion (from 2001 to 2016)**.

**References**	**Cells and samples**	***H. pylori* strains**	**Methods**	**Time after Hp infection**	**Implications**
Liu et al., 2012	AGS cells	J99 and and its isogenic nudA mutant	FISH, TEM	12 h	VacA were closely associated with intracellular *H. pylori*
Chu et al., 2010	AGS cells and MKN45 cells	238 and its isogenic babA, cagA and vacA mutant, 917, 1076, 1024, 43504, J99	CLSM, TEM	6–12 h	The autophagic vesicles induced by *H. pylori* are the sites of replication and also of the degradation of the replicating bacteria after fusion with lysosomes
Ito K. et al., 2008	Huh7 cells and AGS cells	43504 and 401C	EM, TEM	24 h	*H. pylori* adhered to and invaded into hepatocytes more efficiently than into gastric epithelial cells depending on the virulent factors
Terebiznik et al., 2006	AGS cells and CHO-II a cells	49503 and its isogenic vacA mutant	RFM, CLSM	48 h	Ability of *H. pylori* to invade AGS cells was independent of the VacA and VacA enhanced long-term survival of the bacteria
Kwok et al., 2002	AGS cells, HEp-2 cells and HeLa cells	P1, P12, 26695, J99, P49	TEM, SEM, CLEM	12 h	Entry of *H. pylori* into AGS cells occurs via a zipper-like mechanism
Amieva et al., 2002	AGS cells, Caco-2 cells and MDCK cells	G27 and its isogenic vacA– and cagA– mutants	VM, IF, CLSM	within 45 min	*H. pylori* enter and survive within multivesicular vacuoles of epithelial cells
Petersen et al., 2001	AGS cells	AF4 and its isogenic vacA mutant, G27, 51932	TEM	3–24 h	VacA improves the intracellular survival of *H. pylori* within AGS cells
Zhang et al., 2015	AGS cells	43504, 26695, SS1, clinicalisolates	TEM	4–6 h	*H. pylori* invasive ability and disease severity have a positive correlation
Lozniewski et al., 2003	AGS cells;Gastric ucosa samples of human gastric xenografts in nude mice	UA948 and UA948 fucTa_a, 26695 and 26695fucTa_b, UA1111 and UA1111fucT2_	TEM	6 h; Whitin 2weeks	LeX may be involved in *H. pylori* internalization
Björkholm et al., 2000	the human epithelial cell, HEp-2	88-23, CCUG 17874 and its isogenic vacA mutant	TLP	1–6 h	H. pylori has the potential to invade epithelial cells actively
Zhang et al., 2007	AGS cells, SGC-7901 cells, MDCK cells	X47, SS1 and its isogenic vacA mutant, 88-3887 and its isogenic cagA mutant	EM, PCR	5 h	CagA and VacA are not related to the ability of invasion and adhesion of H. pylori in different cell lines in vitro
Wang et al., 2016	GES-1 cells	clinicalisolates	PCR, E-test, K–B method	7 or 10 days	*H. pylori* invasion of the gastric epithelia might play a role in eradicaton failure
Vázquez-Jiménez et al., 2016	AGS cells	clinicalisolates	-	6 h	there was no correlation between adherence pattern and invasiveness
Ozbek et al., 2010	Gastric biopsy specimens of patients with gastric discomfort	clinicalisolates	EM, IHC	–	*H. pylori* within the membrane-bounded vacuoles of both the gastric epithelial cells and the lamina propria
Semino-Mora et al., 2003	Biopsy specimens of patients with metaplasia, dyspepsia and neoplasm	clinicalisolates	CSLM, ISH, IHC, CLSM, TEM	–	*H. pylori* penetrates normal, metaplastic and neoplastic gastric epithelium in vivo
Ito K. et al., 2008	Stomach and gastric lymph nodes of patients with H. pylori infection	clinicalisolates	Real-time PCR, IHC	–	*H. pylori*-induced gastric epithelial damage allows the bacteria to invade the lamina propria and translocate to the gastric lymph nodes

Cell lines: AGS cells (human gastric adenocarcinoma epithelial cell line); MKN45 (human gastric carcinoma cell line); Huh7 cells (human hepatocellular carcinoma cell line); CHO-II a cells (Chinese hamster ovary cells stably transfected with Fc_IIa receptors); HEp-2 cells (human laryngeal carcinoma cell line); HeLa cells(Human cervix carcinoma cell line); Caco-2 cells(human colon adenocarcinoma cell line); MDCK (the canine kidney tubular epithelial line); SGC-7901 cells (human gastric cancer cell line).

^*^Gentamicin protection test for all cell experiments.

CLSM, Confocal laser scanning microscopy; MEFs, Murine embryonic fibroblasts; FISH, Fluorescence in situ hybridization; ISH, In situ hybridization; TEM, Transmission electron microscopy; EM, Electron microscopy; SEM, Scanning electron microscope; VM, Video microscopy; TLP, Time-lapse photography; RFM, Ratiometric fluorescence microscopy; CSLM, Concurrently standard light microscopy; Real-time PCR, Real-time polymerase chain reaction; IF, Immunofluorescence; IHC, Immunohistochemistry.

Invasive ability also varies with the host cell type and *H. pylori* strain, with a rate of approximately 0.01–0.1% of inoculated bacteria (Rautelin et al., 1995; Burridge and Chrzanowska-Wodnicka, 1996; Amieva et al., 2002; Dubois and Borén, 2007; Ito K. et al., 2008; Chu et al., 2010). Zhang et al. (2007) indicated that the strain 88-3887 can invade cells better than strains SS1 and X47, and each strain had the highest invasive ability in SCG-7901 cells. As *H. pylori* typically does not possess the ability to enter cells freely, most of the bacterial cells are internalized into the cytoplasm via endocytosis (Ozbek et al., 2010). A study from Stanford University (Amieva et al., 2002) showed that due to the innate immune response, cells can form phagocytic vesicles that wrap around *H. pylori* after cellular entry, even though the vesicles were immature. The encapsulated *H. pylori* cells were not swallowed or digested; instead, the vesicle acted as a protective barrier, allowing the microbes to evade the immune response.

**Figure 1:**
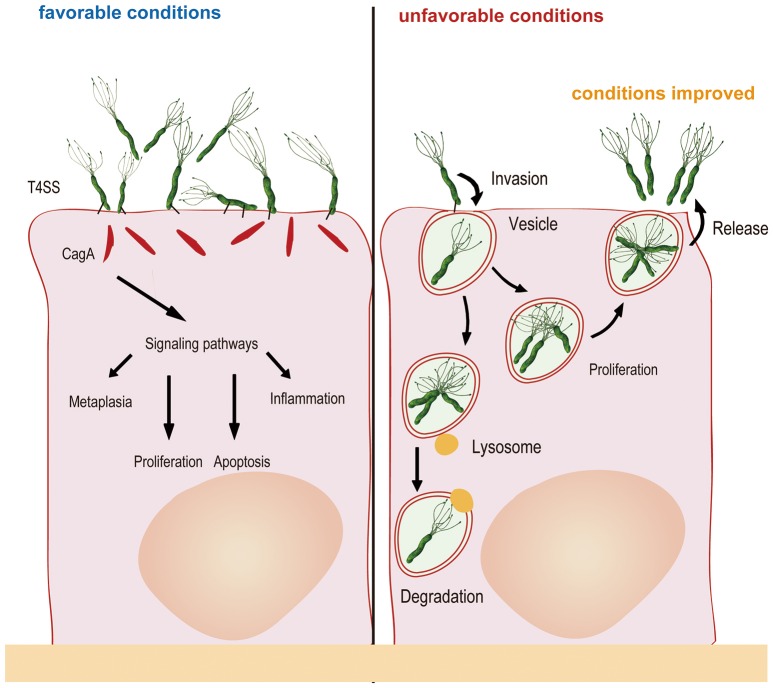
**Generally, ***H. pylori*** adheres to gastric mucosa epithelial cells via the outer membrane protein and injects CagA into host cells via a type IV secretory system, resulting in changes in cytokine signaling and cell cycle control**. When the external environment changes and becomes unfavorable, *H. pylori* invades the epithelial cells and multiplies in double-layer membrane vesicles to seek refuge. After internalization, autophagosomes form to degrade some of the ingested bacteria. Once the external environment becomes favorable, undegraded *H. pylori* is released from host cells into the external environment for recolonization.

These investigators also used differential immune fluorescence staining and live video microscopy to further investigate the physiological activities of internalized *H. pylori* and found that internalization occurred within 45 min of bacterial attachment to the cell surface. The bacterial cells then entered vacuoles and remained viable for at least 48 h. However, replication of the internalized *H. pylori* cells was not observed in the study, perhaps because of the short monitoring time and because the intracellular milieu was not suitable for bacterial replication (Amieva et al., 2002). Nonetheless, a most recent study found that *H. pylori* could proliferate in cells: two cells lines (AGS and MKN45) were evaluated in gentamicin protection experiments, revealing that *H. pylori* could proliferate after entering the cells, with the maximum number of bacterial cells observed after 6–12 h (van der Wouden et al., 1999; Rokkas et al., 2009; Chu et al., 2010). Zhang et al. (2015) also observed this phenomenon in their study, whereby *H. pylori* attached to the cell membrane and combined with cellular microvilli after 2.5 h of infection. After invasion, double-layer membrane vesicles surrounded the *H. pylori* cells (4 h). At 6 h after infection, invasion reached a peak, with many bacteria observed in the cytoplasm. Bacterial adhesion, invasion, division and lysis were observed after 12 h, and most of the intracellular bacterial cells had lysed at 24 h. Another study confirmed that autophagosomes form to degrade ingested *H. pylori* through the lysosomal killing mechanism at 24 h after invasion (Chu et al., 2010). *H. pylori* possesses sophisticated mechanisms. For example, the bacterium seeks refuge inside host cells when the external environment changes and becomes unsuitable for survival; once the external conditions become suitable for survival, undegraded *H. pylori* is released from host cells into the external environment at the appropriate time. *In vitro* experiments have shown that when gentamicin was removed, viable *H. pylori* repopulates the extracellular environment, in parallel with a decrease in the number of intravacuolar bacteria (Amieva et al., 2002). Regardless, how *H. pylori* exits the intracellular space remains unclear. It is possible that viable bacteria are released as infected cells die. Vacuoles containing live *H. pylori* resemble multivesicular bodies, which, in some cell types, are capable of exocytosis and thus release their contents into the extracellular medium (Amieva et al., 2002). In brief, the number of bacteria in the cell is maintained through a dynamic process involving invasion, proliferation, apoptosis and release (Figure 1).

To some extent, these findings provide a clear explanation for repeated infection by *H. pylori*: bacterial cells could escape the immune response of epithelial cells after invasion, and the amount and pathogenicity of the bacteria do not decrease during this process. Such a process might be a strategy for *H. pylori* to escape immune surveillance and remain alive *in vivo* (Amieva et al., 2002; Dubois and Borén, 2007), and it contributes to the complication of eradication. To determine whether *H. pylori* internalization plays a role in treatment failure, one study (Wang et al., 2016) carried out in our laboratory applied an invasion assay to evaluate the levels of *H. pylori* invasion of GES-1 cells. The results showed that the internalization levels of the failing strains were higher than those of the successful strains. However, there is no consensus regarding whether the level of internalization is related to antibiotic resistance, with some studies considering that resistant strains are associated with significantly higher internalization activity than susceptible strains (Lai et al., 2006) but Wang et al. (2016) reporting no evidence of this. Regardless of the correlation, once therapy fails, more consideration should be given to *H. pylo*ri internalization activity.

### Invasion mechanisms

There is increasing research on the invasion mechanism of invasive bacteria, and invasion could proceed by direct engagement of surface host cell receptors or by direct translocation of bacterial proteins into the host-cell cytosol that promote rearrangements of the plasma membrane architecture and induce pathogen engulfment. The former is the “zipper” mechanism, and *Yersinia* and *Listeria* invade cells in this manner. The latter is the “trigger” mechanism, which is used by such bacteria as *Escherichia coli*, and *Shigella, Salmonella* (Pizarro-Cerdá and Cossart, 2006). The invasion mechanism of *H. pylori* remains unclear to date. Kwok et al. (2002) found that *H. pylori* invasion of AGS cells involves close contact with microvilli on the cell membrane and activation of tyrosine phosphorylation signals. Su et al. (1999) reported that H. *pylori* enters cultured AGS cells via the beta 1 integrin receptor in a tyrosine kinase-dependent manner. Other researchers report that *H. pylori* enters cells via receptor-mediated endocytosis, which requires cytoskeletal rearrangement (Evans et al., 1992; Birkness et al., 1996). Given the close relationship between beta 1 integrin, the cytoskeleton and the pathogenicity of internalized *H. pylori*, Ito K. et al. (2008) used a beta 1 integrin antibody to block the binding of *H. pylori* to the corresponding receptor on the cell membrane and used cytochalasin D to block intracellular actin polymerization. These experiments were performed to further observe changes in the invasive capability of *H. pylori*, and the results showed that the number of internalized bacteria decreased substantially after application of these factors. Moreover, the effect of cytochalasin D was more effective than that of the beta 1 integrin antibody, though neither was able to completely block invasion, suggesting that other unidentified and unconfirmed pathways mediate the activities of *H. pylori*. Furthermore, the role of bacterial virulence factors in this process is unclear. As mentioned above, *H. pylori* is enveloped by double-layer membrane vesicles after invasion. Amieva et al. (2002) found that vacuoles have the same morphology as late endosomal multivesicular bodies induced by vacuolating cytotoxin A (VacA). The role of VacA in inducing vacuolization is known, and the effect of VacA in *H. pylori* invasiveness has also been reported. Some previous studies (Amieva et al., 2002; Terebiznik et al., 2006; Zhang et al., 2007; Chu et al., 2010) showed that VacA did not influence the cell-invasion capability of *H. pylori*. Although the bacterial cells were found in vacuoles formed through a VacA-dependent process, the researchers proposed that VacA only affects the survival and multiplication of internalized *H. pylori*. However, an *in vitro* study revealed increased internalization in a strain producing the vacuolating cytotoxin compared to an isogenic VacA knockout mutant (Björkholm et al., 2000). Petersen et al. (2001) also found that the vacuolating cytotoxin of *H. pylori* can improve its intracellular survival in AGS cells. More remarkably, VacA could disrupt autophagy during chronic infection, thereby resulting in a failure to clear the bacteria to promote intracellular survival (Raju et al., 2012). This is different from our previous understanding that autophagy can be induced as an innate defense mechanism to protect against *H. pylori* infection (Orvedahl and Levine, 2009). This phenomenon is mainly due to the length of exposure time. During initial infection, bacterial load and levels of VacA may be low, and host cell autophagy plays an important role in clearance of bacteria; During chronic infection, prolonged exposure to VacA will make immature autophagosomes form (Raju et al., 2012). Ito K. et al. (2008) also used two types of *H. pylori* differing in virulence to compare invasive ability and found better virulence and invasiveness in the strain harboring the cag pathogenicity island (PAI), Vac A, OipA, and BabA isogenes. Based on this evidence, virulence factors also play a role in cell invasion, even though the role of each factor in internalization remains unclear and awaits clarification by experiments involving isogenic mutants. Conversely, a study from China found the opposite result: invasive ability was associated with the VacA mid-region and not with cytotoxin-associated gene A (Cag A), Cag A-EPIYA, or Cag E (Zhang et al., 2015). Therefore, the role of bacterial virulence factors requires more research. In addition, as Nudix enzymes are typically involved in bacterial invasion of eukaryotic cells (Maki and Sekiguchi, 1992), some authors speculate that *H. pylori* invasin may play a role in the entry of the bacterium into epithelial cells because the Nudix hydrolase NudA can degrade toxic substances induced during invasion (Lundin et al., 2003). Originally, Lundin et al. (2003) found no quantifiable differences in invasion frequency for the *H. pylori* NudA J99 mutant compared to WT when using a gentamicin protection assay. Liu et al. (2012) confirmed the previous report, utilizing FISH and an ultrastructural approach to make changes to the “classical” gentamicin protection assay and demonstrating significantly more intracellular and fewer membrane-bound *H. pylori* in WT-infected AGS cells than in cells infected with a mutant carrying a ΔnudA allele. These observations indicated that NudA plays a biologically significant role in *H. pylori* entry into host cells and emphasized that the sensitivity of the “classical” assay may not be sufficient to demonstrate differences in invasion capacity. This lack of sensitivity may be due to the fact that complete killing of extracellular bacteria is rarely achieved (Amieva et al., 2002).

Another concern is the invasiveness of *H. pylori*. As mentioned above, binding of the bacterium to the cell membrane is needed prior to internalization, and it is unclear whether there is any association between adherence pattern and invasiveness. Vázquez-Jiménez et al. (2016) found that *H. pylori* strains exhibiting localized adherence would be more reactive toward gastric epithelial cells compared to the strains exhibiting diffused adherence; this would result in more damage and more proinflammatory signals but has no significant association with invasiveness. However, another report showed a specific ratio of invasive and adherent *H. pylori* (Demirel et al., 2013). Zhang et al. (2015) found no significant differences among various strains with regard to the ratio of invasion and adhesion, a finding that shows that the amount of adherent bacteria rather than the bacterial strain determines the final amount of invasive bacteria. Adhesion is the single major determinant for cell invasion by *H. pylori*, and many researchers have performed various invasion experiments to further investigate this process. In an *in vitro* study, Kwok et al. (2002) found the level of *H. pylori* entry into epithelial cells to be within the same range as that of other known invasive pathogens, such as *Salmonella enterica, Escherichia coli, Yersinia enterocolitica*, and *Neisseria gonorrhoeae*. They also observed greater *H. pylori* uptake by AGS cells than by HeLa or HEp-2 cells, indicating that uptake mainly depends on the host cell type.

### Association with pathogenesis

The pathogenicity of bacteria that colonize the human mucosa is influenced by their capacity to invade and survive within epithelial cells. However, whether *H. pylori* internalization affects pathogenicity remains controversial. An *in vivo* study showed a positive correlation between the invasive ability of *H. pylori* and disease severity, and the average invasion rates of *H. pylori* strains found in gastric cancer and ulcers were higher than the rate of strains found in gastritis (Zhang et al., 2015). Moreover, many studies have confirmed that internalized *H. pylori* is pathogenic and able to express the virulence proteins Cag A and Vac A (Semino-Mora et al., 2003; Dubois and Borén, 2007; Necchi et al., 2007; Ito T. et al., 2008; Chu et al., 2010). Internalized *H. pylori* can also activate the nuclear factor kappa-light-chain-enhancer of activated B cells (NF-κB) signaling pathway and induce interleukin-8 (IL-8) secretion, which suggests that invasion by this bacterium might be an important strategy in the development of *H. pylori*-associated diseases (Zhang et al., 2015). In addition, *in vivo* and *in vitro* studies show an association between internalized *H. pylori* and cell damage as well as cell disintegration (el-Shoura, 1995; Wilkinson et al., 1998). Ito T. et al. (2008) even found that *H. pylori*-induced gastric epithelial damage allows the bacterial cells to invade the lamina propria and translocate to the gastric lymph nodes, which may chronically stimulate the immune system. Additionally, the bacterial cells, alive or not, captured by macrophages may contribute to the induction and development of *H. pylori*-induced chronic gastritis.

## Conclusions

*H. pylori* is the main pathogenic bacteria of chronic active gastritis, peptic ulcers, gastric mucosa-associated lymphoid tissue lymphoma, and gastric cancer. This bacterium seeks refuge inside cells when the external environment is not suitable, and it can also complete an entire biological cycle, including proliferation and apoptosis, within gastric epithelial cells. Furthermore, once the external conditions become suitable, undegraded *H. pylori* cells leave the host cell and can be released to infect other cells and cause repeated infection. The above findings suggest that invasion plays an important role in the induction of disease, immune escape and chronic infection. Moreover, these results provide a new direction for research on the pathogenic mechanism of *H. pylori*-associated gastric diseases. The latest consensus recommendations for treating *H. pylori* infection are non-bismuth quadruple therapy and traditional bismuth quadruple therapy as first-line strategies (Fallone et al., 2016). To date, studies on the reasons for eradication failure have concentrated on antibiotic resistance. However, *H. pylori* internalization provides a new research focus. The antibiotics in the recommended regimens include both cell membrane-penetrating antibiotics, such as clarithromycin or metronidazole, and antibiotics that do not penetrate the cell membrane, such as amoxicillin. Increasing the concentration and treatment time of cell membrane-penetrating antibiotics that effectively penetrate epithelial cells to kill intracellular *H. pylori* might help in achieving complete eradication.

## Author contributions

YH, QW, DC, WX, and NL contributed equally to this review.

## Disclosures

Language has been improved by the company named AJE.

### Conflict of interest statement

The authors declare that the research was conducted in the absence of any commercial or financial relationships that could be construed as a potential conflict of interest.
